# Optimization, Equilibrium and Kinetic Modeling of Methylene Blue Removal from Aqueous Solutions Using Dry Bean Pods Husks Powder

**DOI:** 10.3390/ma14195673

**Published:** 2021-09-29

**Authors:** Giannin Mosoarca, Simona Popa, Cosmin Vancea, Sorina Boran

**Affiliations:** Faculty of Industrial Chemistry and Environmental Engineering, Politehnica University Timisoara, Bd. V. Parvan No. 6, 300223 Timisoara, Romania; giannin.mosoarca@upt.ro (G.M.); sorina.boran@upt.ro (S.B.)

**Keywords:** optimization, Taguchi method, methylene blue, adsorption, equilibrium modeling, kinetics, thermodynamics

## Abstract

In this research, dry bean pods husks (DBPH) were used as an adsorbent material after minimum processing (without chemical substances consumption and without thermal treatment) to remove methylene blue from aqueous solutions. The adsorbent surface characteristics were investigated using SEM and FTIR analysis. For maximum removal efficiency, several parameters that influence the dye adsorption were optimized using the Taguchi method. Equilibrium and kinetic modeling, along with thermodynamic studies, were conducted to elucidate the adsorption mechanism. Taguchi experimental design showed that the factor with the highest influence was the adsorbent dose, with a percent contribution established by the ANOVA analysis of 40.89%. Langmuir isotherm and pseudo-second order kinetic model characterizes the adsorption process. The maximum adsorption capacity, 121.16 (mg g^−1^), is higher than other similar adsorbents presented in scientific literature. Thermodynamic parameters indicate a spontaneous, favorable and endothermic adsorption process, and their values show that physical adsorption is involved in the process. The obtained results, and the fact that adsorbent material is inexpensive and easily available, indicate that DBPH powder represents an effective absorbent for treating waters containing methylene blue. Additionally, the Taguchi method is very suitable to optimize the process.

## 1. Introduction

Dyes are an important category of compounds widely used in many industries: textile, dye, plastic, paper, leather, rubber and cosmetics [1,2,3,4,5,6,7,8,9]. The residual effluents can contain variable amounts of dyes and without prior treatment, before discharge, they can cause serious problems both to the environment and human health [2,3,6,7,9,10,11].

Methylene blue (MB) dye is used in many industrial fields as well as in medicine. Even if it is not very toxic and dangerous, this compound can cause negative effects on human health: respiratory problems, vomiting, increased heart rate, cyanosis, eye irritation, nausea, methemoglobinemia, diarrhea and jaundice [2,9,11,12,13,14,15,16,17]. Therefore, its removal from process effluents is absolutely required.

Unlike other methods used to remove dyes from wastewater (coagulation, precipitation, ion exchange, membrane processes, chemical oxidation, electrochemical processes, biodegradation), adsorption has several essential advantages such as high efficiency, ease of operation, flexibility and low costs [1,3,4,6,7,8,9,10,12,13,14,15,16,17,18].

The actual trend is to identify various new low-cost adsorbents such as natural materials, industrial wastes, agricultural wastes and bioadsorbents [1,3,8,12,13,14].

Another major advantage is the fact that the adsorption process can be easily optimized and modeled [19,20,21,22,23,24]. 

The Taguchi method is used to optimize various processes without increasing costs. It allows understanding the effect of variable process parameters in a small number of experimental tests, and the results obtained lead to improved process performance [19,21,22,23,24,25,26]. The Taguchi method uses an orthogonal array that distributes the variables in a balanced way, and the experimental results are converted into a signal-to-noise ratio (S/N). The optimal value of the process parameters is given by the highest S/N ratio [19,21,22].

Common bean (*Phaseolus vulgaris* L.) is an important legume that grows in subtropical and temperate regions. Its seeds are a very important source of food for large parts of the population due to its nutritional properties (high source of dietary fiber and proteins, low level of lipids), low cost and long-term storage possibility. Almost 27 million tones of beans are produced annually in the world [27,28,29]. If common bean is grown for dry beans, a significant amount of dry pod husks results after harvesting and separation of the grains. 

The aim of the present paper was to use this product, after minimum processing (without chemical substances consumption and without thermal treatment), to remove methylene blue dye from aqueous solutions by adsorption. Scanning electron microscopy (SEM) and Fourier transform infrared spectroscopy (FTIR) were carried out to study the adsorbent surface characteristics. For maximum removal efficiency the parameters that influenced the adsorption process were optimized by using the Taguchi method. For a description of the adsorption process, equilibrium, kinetics and thermodynamic parameters were calculated and discussed.

## 2. Materials and Methods

### 2.1. Adsorbent Preparation and Characterization

Dry bean pods husks (DBPH) were provided by a local agricultural producer from Cerneteaz village, Romania. The husks were first washed using distilled water, then dried at room temperature for three days and then at 90 °C for 24 h. The next operations to which the material was subjected were grinding (with an electric mill), passing over a sieve (with a mesh diameter of 2 mm), washing with distilled water (for turbidity and color removal) and drying at 105 °C for 5 h.

SEM analysis (Quanta FEG 250 microscope, at 1600× magnitude) was used to study the surface morphology of the adsorbent material. Identification of functional groups of the adsorbent was performed by FTIR spectroscopy (Shimadzu Prestige-21 FTIR spectrophotometer). The FTIR spectrum was recorded after the solid adsorbent sample was ground with IR transparent potassium bromide (KBr) and then pressed into a pellet.

### 2.2. Taguchi Experimental Design

The Taguchi (L27) orthogonal array was used to establish the optimum conditions for the dye removal by adsorption. The effect of five factors, at three levels, on the removal efficiency of dye was studied. Table 1 presents the controllable factors and their levels, which were used in the Taguchi design.

The Taguchi approach converted the obtained experimental results into a signal-to-noise (S/N) ratio, which was used to analyze the experiment quality and the validity of the result. The terms “signal” and “noise” represent the desirable value (mean) and the undesirable value (standard deviation) for the output characteristic, respectively. To evaluate the experimental results, the highest adsorption efficiency was considered. In analyzing the signal-to-noise ratio of the Taguchi method, the “larger-the-better” option (Equation (1)) was chosen [19,30,31,32]:

(1) $$\frac{S}{N}=-{log}_{10}\left[\frac{1}{n}\sum_{i=1}^{n}{\left(\frac{1}{y_{i}}\right)}^{2}\right]$$

where: *n* represents the repetitions number under similar experimental conditions, and *y_i_* represents the experimental response. 

The S/N ratio was calculated and analyzed using the Minitab19 software.

To establish the percentage contribution of each factor to the efficiency of MB removal and to evaluate the results of the Taguchi model, an analysis of variance (ANOVA-General Linear Model) was used [19,30,31,32,33]. The necessary calculations were performed with the Minitab 19 software.

### 2.3. Adsorption Experimental Studies 

The adsorption studies were performed at constant mixing intensity, in Erlenmeyer flasks, using 50 mL of MB solution together with the adsorbent material. The pH adjustment was realized with dilute solutions of NaOH and HCl (0.1 N). The dye concentration was determined by a UV–VIS spectrophotometer at 664 nm wavelength. 

The adsorption capacity at equilibrium, (*q_e_*), and at time *t*, (*q_t_*), were calculated with Equations (2) and (3), while the dye removal percentage *R(%)* was calculated with Equation (4) [4,9,10,12,14]:

(2) $$q_{e}=\frac{(C_{0}-C_{e})\cdot V}{m}$$

(3) $$q_{t}=\frac{(C_{0}-C_{t})\cdot V}{m}$$

(4) $$R(\%)=\frac{\left(C_{0}-C_{e}\right)}{C_{0}}\times 100$$

where: *C*_0_ represents the initial MB concentration (mg L^−1^), *C_e_* represents the MB equilibrium concentration (mg L^−1^), *C_t_* represents the MB concentration at time *t* (mg L^−1^), *V* represents the solution volume (L) and *m* represents the mass of adsorbent (g).

### 2.4. Equilibrium and Kinetic Modeling

Adsorption isotherms are very important for describing the solid-liquid adsorption process. The Langmuir and Freundlich isotherms were used to investigate the adsorption behavior. Their equations are presented below:

(5) $$\mathrm{Langmuir}\;\mathrm{isotherm}:\;q_{e}=\frac{q_{m}\cdot K_{L}\cdot C_{e}}{1+K_{L}\cdot C_{e}},$$

(6) $$\mathrm{Freundlich}\;\mathrm{isotherm}:\;q_{e}=K_{F}\cdot C_{e}^{1/n_{F}},$$

where: *q_m_* represents the maximum absorption capacity (mg g^−1^), *K_L_* represents the Langmuir constant, *K_F_* represents the Freundlich constant and 1/*n_F_* represents an empirical constant indicating the adsorption intensity [34,35,36,37,38].

A lot of useful information on the mechanism and efficiency of adsorption used to design an industrial treatment plant is provided by kinetics studies. The pseudo-first-order and pseudo-second-order models were used to model the experimental data.

(7) $$\mathrm{Pseudo-first-order}\;\mathrm{model}\;\mathrm{equation}:\;q_{t}=q_{e}(1-{exp}^{-k_{1}\cdot t}),$$

(8) $$\mathrm{Pseudo-second-order}\;\mathrm{model}\;\mathrm{equation}:\;q_{t}=\frac{k_{2}\cdot t\cdot q_{e}^{2}}{1+k_{2}\cdot t\cdot q_{e}},$$

where: *k*_1_ represents the pseudo-first-order model rate constant, and *k*_2_ represents the pseudo-second-order model rate constant [34,35,36,37,38].

To establish the best-fitting kinetic and equilibrium models, the values of determination coefficient (*R*^2^), sum of square error (*SSE*), chi-square (*χ*^2^) and average relative error (*ARE*) were determined with the equations described below [37]. The higher value for *R*^2^ and the smaller values for *SSE*, *χ*^2^ and *ARE* were taken into account when choosing the most suitable models.

(9) $$R^{2}=1-\frac{\sum_{i=1}^{n}{\left(y_{i,exp}-y_{i,mod}\right)}^{2}}{\sum_{i=1}^{n}{\left(y_{i,exp}-\bar{y_{i,exp}}\right)}^{2}}$$

(10) $$SSE=\sum_{i=1}^{n}{\left(y_{i,exp}-y_{i,mod}\right)}^{2}$$

(11) $$\chi^{2}=\sum_{i=1}^{n}\frac{{\left(y_{i,exp}-y_{i,mod}\right)}^{2}}{y_{i,mod}}$$

(12) $$ARE=\frac{100}{n}\sum_{i=1}^{n}\left|\frac{y_{i,exp}-y_{i,mod}}{y_{i,mod}}\right|$$

where: *y_i,exp_* represents the independent variable experimental value, *y_i,mod_* represents the modeled value, $\bar{y_{i,exp}}$ represents the observed values mean o and *n* is the information total number.

### 2.5. Thermodynamic Parameter Determination

The data of methylene blue adsorption, at different temperatures (285, 296 and 306 K), were used to determine Gibbs free energy change, enthalpy change and entropy change, according to the following equations [37,39,40]:

(13) $$\Delta G^{0}=-RTlnK_{L}$$

(14) $$lnK_{L}=\frac{\Delta S^{0}}{R}-\frac{\Delta H^{0}}{RT}$$

where: *R* represents the universal gas constant, *K_L_* represents the Langmuir constant and *T* represents the absolute temperature.

### 2.6. Desorption Experimental Studies

In the desorption studies, the MB-loaded adsorbent was mixed with various desorption agents: 0.1 N HCl, 0.1 N NaOH and distilled water.

The desorption percent *D*(*%*) of dye was calculated with Equation (15):

(15) $$D(\%)=\frac{m_{d}}{m_{a}}\times 100$$

where: *m_d_* represents amount of dye liberated by desorbing agent, and *m_a_* represents amount of dye adsorbed on adsorbent.

## 3. Results and Discussion

### 3.1. Adsorbent Material Characterization

**Figure 1:**
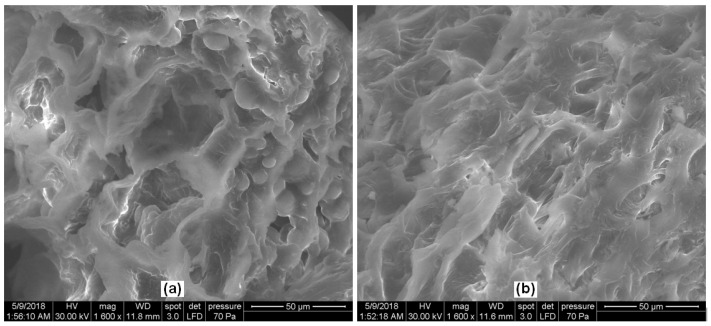
Scanning electron microscopy images of DBPH powder: (**a**) before and (**b**) after MB adsorption.

SEM analysis of the adsorbent material showed the presence of irregular pores with different shapes and sizes on its surface (Figure 1a). After adsorption, the surface was modified, and the pores were covered by dye molecules (Figure 1b).

**Figure 2:**
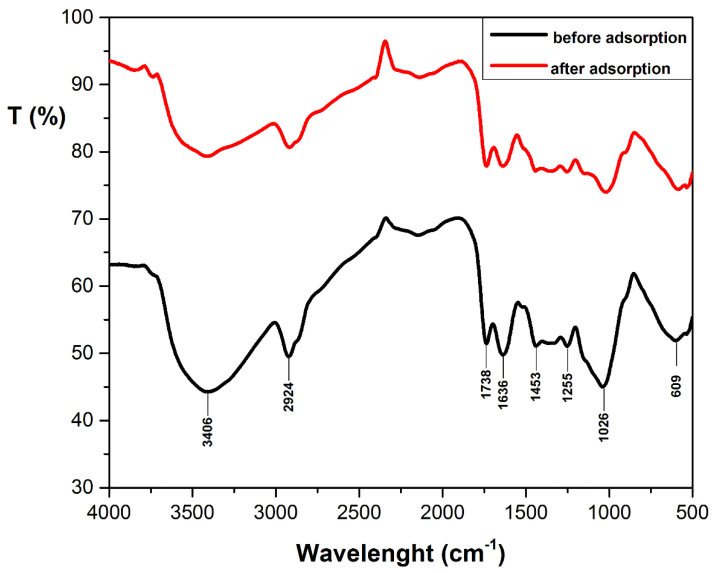
FT-IR spectrum of DBPH powder.

FTIR spectroscopy was used to identify the presence of different functional groups on the surface of adsorbent. The FTIR spectra illustrated in Figure 2 suggest cellulose and hemicellulose as main components. The differences between the peak’s wavenumber before and after adsorption are less than 10 cm^−1^, indicating an adsorption mechanism that could include physical interaction or ion-exchange mechanism [41].

The specific peaks of the main functional group are: 3406 cm^−1^—strong absorption peaks of O-H stretching vibration [42]; 2924 cm^−1^—the peak belongs to -CH_2_ groups of cellulose [43]; 1738 cm^−1^—C=O stretching vibration of carboxylic groups of hemicellulose [44]; 1636 cm^−1^—O-H bending vibration of water sorption characteristics of cellulose [45]; 1453 cm^−1^—the peak belongs to the bending of -CH groups of cellulose [43]; 1255 cm^−1^—C–O stretching and CH or OH bending of hemicellulose structures [40,46]; 1026 cm^−1^—C–O, C–O–H, C–O–C, C–C, ring stretching vibration in cellulose and hemicellulose [47]; 609 cm^−1^—the bending modes of aromatic compounds of cellulose [48].

### 3.2. Optimization of Adsorption Parameters

Five controllable factors at three levels were used in the Taguchi design to estimate the optimum conditions for MB adsorption. Table 2 shows the L27 orthogonal array and results obtained after each run. Using the rank of S/N ratio, along with total increments (delta values), the order of the controllable factors’ significance was determined (Table 3). The delta value measures the magnitude of the effect considering the difference between the highest and lowest characteristic average for a controllable factor [19]. The factor that had the greatest influence on the process was the adsorbent dose, while the factor with the least influence was temperature. The optimum conditions of adsorption are also marked in Table 3.

**Figure 3:**
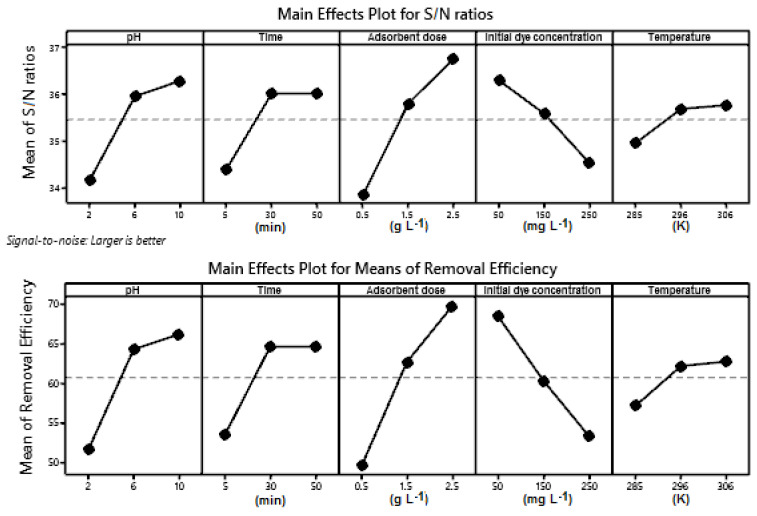
The response curves for the individual effects of dye adsorption parameters on S/N ratio and dye removal efficiency.

Figure 3 shows comparatively the response curves for the individual effects of dye adsorption parameters on the S/N ratio and dye removal efficiency.

The adsorbent dose has a greater influence on process efficiency due to the adsorption surface area and the number of sites available for adsorption increase with the adsorbent material dose [14,36,49]. Another parameter that has a great influence on the process is pH. Dye removal efficiency increases with the increasing of pH in the range 2–10. At lower values of pH the adsorbent surface is positively charged, but with the increase in this parameter the adsorbent surface became negatively charged and favored the electrostatic attraction with MB cations, resulting in a better efficiency [14,36,39]. With the increasing of the initial dye concentration, almost all the adsorption sites on the adsorbents became saturated due to the accumulation of dye molecules on the surface of the adsorbent particle and the removal percentage of the dye decreasing [1]. The impact of contact time was significant at the beginning of the process when a large number of active sites on the adsorbent surface were available for MB adsorption that generated a rapid increase in dye removal efficiency, until the equilibrium was reached after 30 min. The temperature had a lower influence on the dye removing process from the aqueous solution. The increase in temperature reduces the solution viscosity and has a positive effect on the mobility of the dye cations [39].

**Figure 4:**
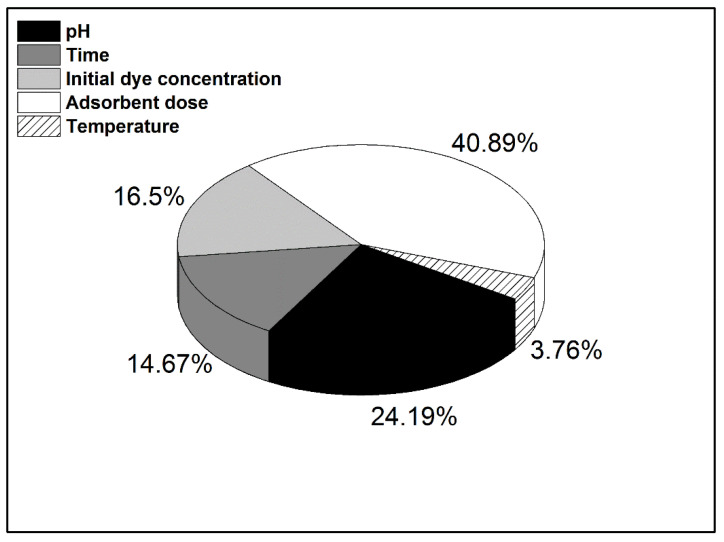
Specific influence of controllable factors on the adsorption process.

**Figure 5:**
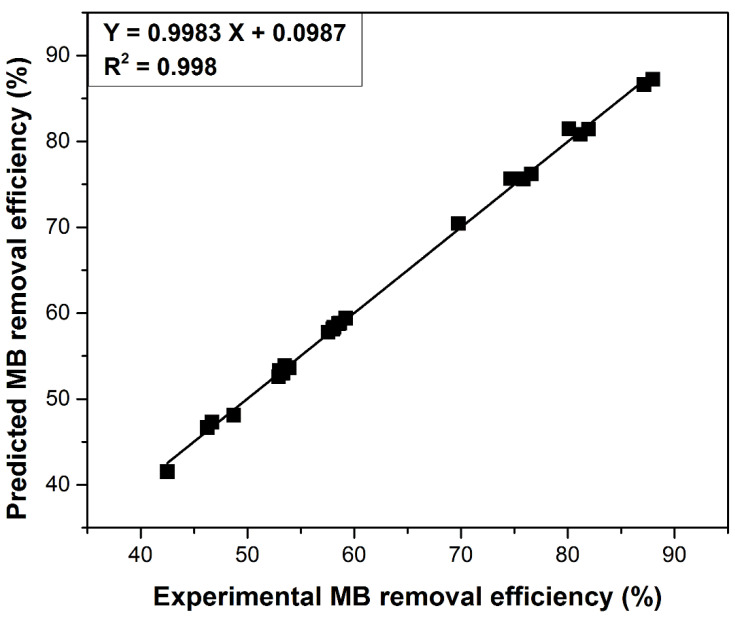
Comparison of experimental and predicted MB removal efficiency.

The order of the controllable factor influence predicted by the Taguchi design was confirmed by analysis of variance (ANOVA-General Linear Model). Figure 4 illustrates the specific influence of each factor on dye removal by adsorption on DBPH powder. Even though the Taguchi experimental design is based on a limited number of experiments, by analyzing the correlation of the predicted MB removal efficiency with experimental results, it can be observed that the accuracy of the Taguchi method prediction was very good (Figure 5).

### 3.3. Equilibrium Modeling

While Langmuir isotherm assumes a monolayer adsorption on a homogeneous surface, Freundlich isotherm considers a multilayer adsorption on a heterogeneous surface, with the sites having different affinity [2,7,19].

**Figure 6:**
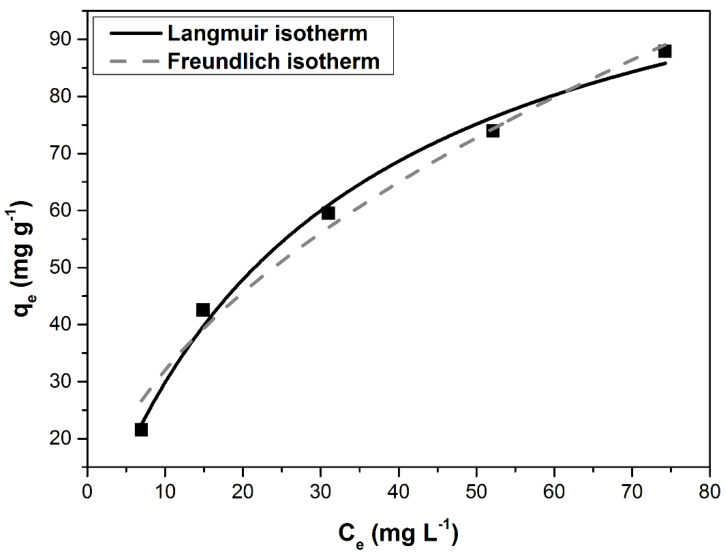
Langmuir and Freundlich isotherms for MB adsorption on DBPH powder.

Both isotherms are comparatively illustrated in Figure 6. The values of the isotherm’s constants, summarized in Table 4, indicate that the adsorption process follows the Langmuir isotherm. The maximum adsorption capacity of 121.16 (mg g^−1^) is comparable to those previously reported in the literature, even higher than other similar adsorbents (Table 5). 

### 3.4. Kinetic Modeling

**Figure 7:**
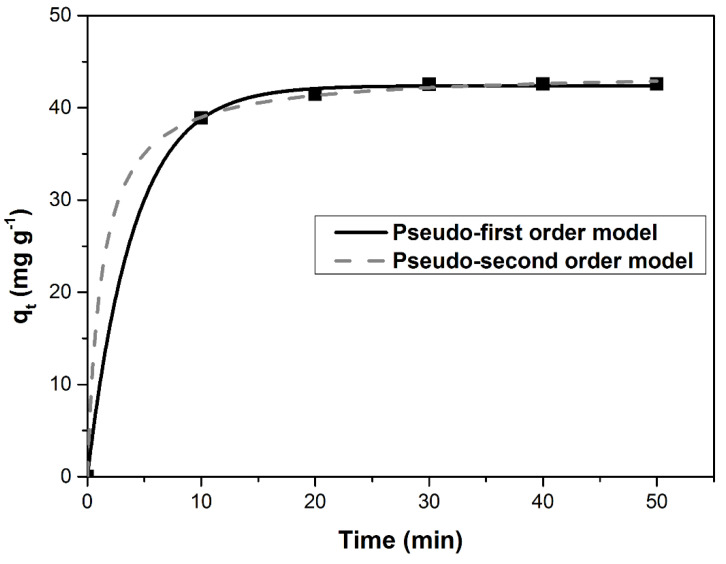
The pseudo-first-order and pseudo-second-order models models tested for MB adsorption on DBPH powder.

The pseudo-first-order and pseudo-second-order models were used in the kinetic study (Figure 7). Kinetic parameters for these models were summarized in Table 6. The pseudo-second-order kinetic model had a higher value for R^2^ and smaller values for SSE, χ^2^ and ARE. Therefore, this model is best suited to describe the adsorption process.

### 3.5. Thermodynamic Parameters

**Figure 8:**
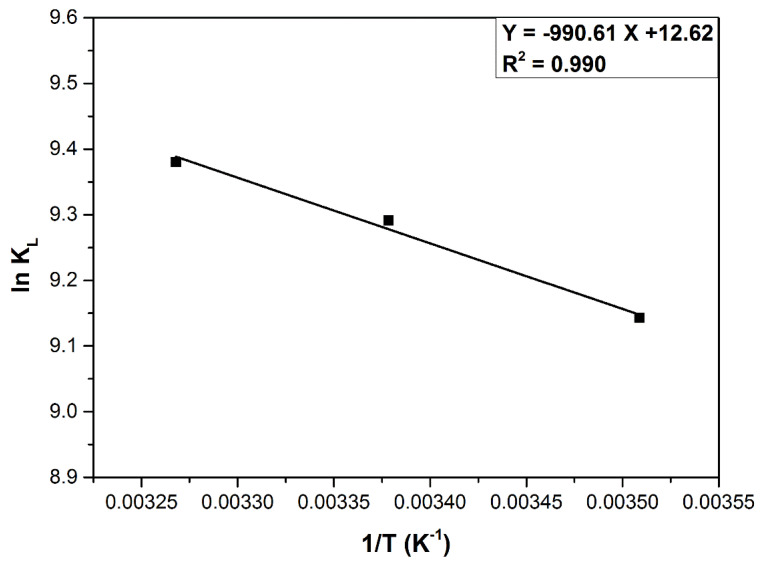
Plot of ln K_L_ vs. 1/T for MB adsorption on DBPH powder.

Thermodynamic parameters, calculated based on the plot presented in Figure 8 and mentioned in Table 7, suggests an endothermic (ΔG^0^ < 0, ΔH^0^ > 0), spontaneous and favorable adsorption process. ΔS^0^ > 0 indicates the affinity of adsorbent material for dye [8,14]. The physisorption is involved in the MB adsorption process when ΔH^0^ < 40 (kJ mol^−1^) [72,73]. In addition, when ΔH^0^ < (20 kJ mol^−1^), the physical adsorption is affected by van der Waals interactions [74]. Generally, ΔG^0^ values ranged between −20 (kJ mol^−1^) up to 0 (kJ mol^−1^) and indicate that physical adsorption is involved, while ΔG^0^ ranging between −80 and −200 (kJ mol^−1^) suggests a possible chemisorption process. The calculated value presented in Table 7 suggests an adsorption based on physisorption and enhanced by a small chemical effect [40].

### 3.6. Desorption Studies

**Figure 9:**
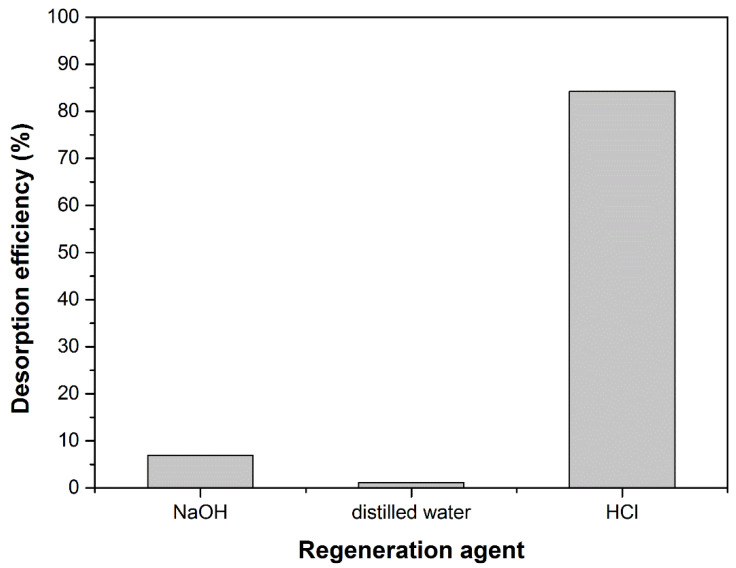
The desorption efficiency in three different media.

In order to consider the regeneration possibility of adsorbent material, the desorption studies were performed in three different media (acid, neutral and basic). Figure 9 shows the desorption efficiency of the tested regeneration agents. The acid was found as being the best desorbing reagent with efficiency of about 85%. 

**Figure 10:**
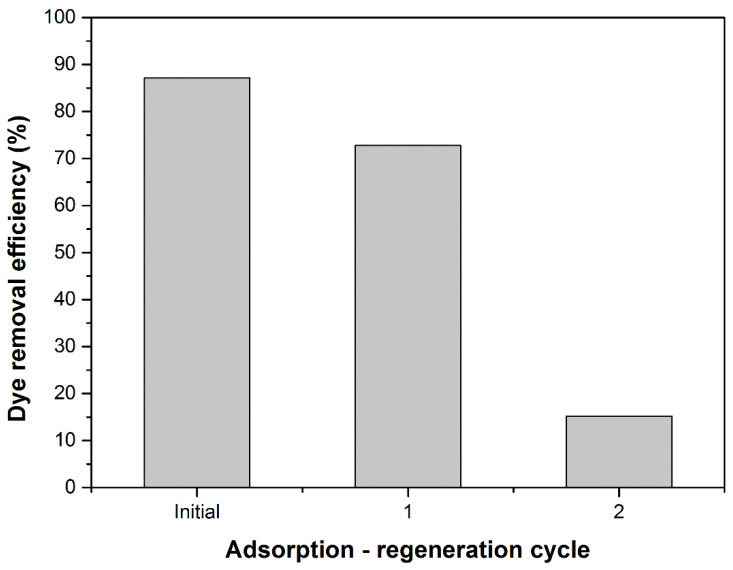
Dye removal efficiency after each adsorption-regeneration cycle.

The efficiency of the adsorption process after several adsorption-regeneration cycles was studied in the next stage of experimental determinations. The results are shown in Figure 10. It can be seen that the efficiency of the dye removal process decreased after each adsorption-regeneration cycle. If initially this parameter was 87% after the first cycle, it decreased to 73%, and after the second cycle it reached 15%. Practically only the first regeneration cycle is recommended, after which the adsorbent material has a lower performance. DBPH powder is cheap and easily available in large quantities; therefore, we consider that regeneration is not mandatory.

## 4. Conclusions

The dye removal efficiency by adsorption on dry bean pods husks powder is influenced by solution pH, contact time, initial dye concentration and adsorbent dose. The Taguchi (L27) experimental design showed that the most influential factor was adsorbent dose (with a percent contribution, established by the ANOVA (General Linear Model) analysis, of 40.89%), followed by pH, initial dye concentration, time and temperature. The accuracy of the Taguchi method prediction was very good (R^2^ = 0.998). Langmuir isotherm and pseudo-second-order kinetic model describe the adsorption process. These kinetic and equilibrium models had greater values for R^2^ and smaller values for SSE, χ^2^ and ARE. The maximum adsorption capacity, 121.16 (mg g^−1^), was higher compared to other similar adsorbents reported in the literature. The thermodynamic parameter values indicate a spontaneous, favorable and endothermic (ΔG^0^ < 0, ΔH^0^ > 0) adsorption process. Furthermore, they denote those electrostatic forces and van der Waals interactions are implied in the physical adsorption process (ΔH^0^ < 20 kJ mol^−1^). The desorption studies performed in three different media (acid, neutral and basic) showed that the acid was the best desorbing reagent with an efficiency of about 85%. The efficiency of the adsorption process after several adsorption-regeneration cycles decreased after each cycle. After the second cycle it reached 15%. The obtained results, and the fact that adsorbent material is cheap and easily available, recommend DBPH powder as an efficient, low-cost adsorbent for methylene blue removal from aqueous media and the Taguchi method as a very suitable adsorption optimization algorithm.

## Figures and Tables

**Table 1:** The controllable parameters and their levels.

Parameter	Level 1	Level 2	Level 3
pH	2	6	10
Time (min)	5	30	50
Adsorbent dose (mg L^−1^)	0.5	1.5	2.5
Initial dye concentration (mg L^−1^)	50	150	250
Temperature (K)	285	296	306

**Table 2:** Experimental layout of L27 orthogonal array and results obtained for removal efficiency and S/N ratios.

pH	Time	Adsorbent Dose	Initial Dye Concentration	Temperature	Dye Removal Efficiency	S/N Ratio
2	5	0.5	50	285	38.81	31.77
2	5	0.5	50	296	42.22	32.51
2	5	0.5	50	306	42.62	32.59
2	30	1.5	150	285	53.95	34.63
2	30	1.5	150	296	58.69	35.37
2	30	1.5	150	306	59.24	35.45
2	50	2.5	250	285	53.35	34.54
2	50	2.5	250	296	58.04	35.27
2	50	2.5	250	306	58.58	35.35
6	5	1.5	250	285	48.67	33.74
6	5	1.5	250	296	52.97	34.48
6	5	1.5	250	306	53.44	34.55
6	30	2.5	50	285	80.12	38.07
6	30	2.5	50	296	87.16	38.80
6	30	2.5	50	306	87.98	38.88
6	50	0.5	150	285	52.94	34.47
6	50	0.5	150	296	57.59	35.20
6	50	0.5	150	306	58.13	35.28
10	5	2.5	150	285	63.67	36.07
10	5	2.5	150	296	69.26	36.80
10	5	2.5	150	306	69.91	36.89
10	30	0.5	250	285	48.73	33.75
10	30	0.5	250	296	53.01	34.48
10	30	0.5	250	306	53.51	34.56
10	50	1.5	50	285	74.66	37.46
10	50	1.5	50	296	81.21	38.19
10	50	1.5	50	306	81.98	38.27

**Table 3:** Signal-to-noise S/N ratios response.

Level	pH	Time	Adsorbent Dose	Initial Dye Concentration	Temperature
1	34.17	34.38	33.85	36.29 *	34.95
2	35.95	36.00	35.80	35.58	35.68
3	36.28 *	36.01 *	36.75 *	34.53	35.76 *
Delta	2.11	1.63	2.90	1.76	0.81
Rank	2	4	1	3	5

* The maximum S/N ratio indicates the optimum condition.

**Table 4:** Langmuir and Freundlich adsorption isotherms constants.

Isotherm Model	Parameters	Value
Langmuir	K_L_ (L mg^−1^)	0.032 ± 0.004
	q_max_ (mg g^−1^)	121.16 ± 7.53
	R^2^	0.9921
	SSE	21.51
	χ^2^	0.41
	ARE (%)	3.89
Freundlich	K_f_ (mg g^−1^)	9.88 ± 1.79
	1/n	0.51 ± 0.04
	R^2^	0.9839
	SSE	44.79
	χ^2^	1.38
	ARE (%)	6.77

**Table 5:** Maximum adsorption capacities for a number of previously similar studied adsorbents.

Adsorbent Material	Maximum Adsorption Capacity (mg g^−1^)	Reference
papaya seeds	555.55	[50]
corncob	417.12	[51]
banana stalks	322.58	[52]
shaddock peel	305.81	[53]
maize silk powder	234.10	[54]
broad bean peels	192.72	[55]
mung bean shell	165.92	[56]
fava beans	140.00	[57]
dry bean pods husk	121.16	This study
coffee husks	90.09	[58]
garlic peel	82.64	[59]
peanut husk	72.13	[60]
peanut hull	68.03	[61]
oiltea shell	64.35	[62]
*Daucus carota* stem powder	55.50	[63]
yellow passion fruit waste	44.70	[64]
rice husk	40.59	[65]
corn husk	30.33	[66]
Bengal gram bean	24.70	[67]
banana peel	20.80	[68]
mucuna beans	19.97	[69]
orange peel	18.60	[68]
raw corn cobs	18.28	[70]
wheat shells	16.56	[71]

**Table 6:** Tested kinetic model parameters.

Kinetic Model	Parameters	Value
Pseudo-first-order	k_1_ (min^−1^)	0.349 ± 0.029
	q_e,calc_ (mg g^−1^)	41.98 ± 0.47
	R^2^	0.9965
	SSE	0.45
	χ^2^	0.013
	ARE (%)	20.55
Pseudo-second-order	k_2_ (g mg^−1^ min^−1^)	0.018 ± 0.001
	q_e,calc_ (mg g^−1^)	43.89 ± 0.16
	R^2^	0.9998
	SSE	0.26
	χ^2^	0.009
	ARE (%)	20.46

**Table 7:** Thermodynamic parameters for MB adsorption on DBPH powder.

ΔG (kJ mol^−1^)			ΔH (kJ mol^−1^)	ΔS (J mol^−1^ K^−1^)
285 K	296 K	306 K	0.99	12.62
−21.66	−22.86	−23.86		

## Data Availability

All the experimental data obtained are presented, in the form of tables and/or figures, in the article.
